# Supplementary material for: Capacity configuration optimization of electricity heat hydrogen regional integrated energy system considering supply-demand uncertainties
Source: PLoS One. 2025 Apr 24;20(4):e0320250. doi: 10.1371/journal.pone.0320250 (PMC12021234; doi:10.1371/journal.pone.0320250)
Supplement: Attachment — Submitted filename: Response to Reviewers.docx [file pone.0320250.s003.docx]

**Response to Reviewer #1：**

1. **Revised in the introduction of the manuscript:**

Therefore, based on the above research, this paper makes the following contributions:

(1) In order to solve the problem of optimal allocation of the electricity heat hydrogen regional integrated energy system, based on typical scenarios, a box-type uncertainty set independent of probability distribution is proposed to describe the uncertainty of wind power and demand, forming robust constraints.

(2) An innovative two-stage robust optimization model is constructed. The first stage of the model is capacity configuration, and the second stage considers the operation in the worst scenario, and adopts min-max-min structure to optimize the configuration. In order to flexibly adjust the degree of conservatism according to the actual demand, the uncertainty adjustment parameter *Г* is introduced into the model. By adjusting the parameter *Г*, the economy and feasibility can be more reasonably balanced, so as to provide higher adaptability for optimization in different scenarios.

(3) In order to further improve the solving efficiency of the model, the model is derived and transformed by combining the column and constraint generation algorithm and the strong duality theory, and the original problem is successfully decomposed into linearized main problem and subproblem, and efficient solution is achieved by alternate solving method.

(4) An integrated energy system in northern China was used to verify the reliability and applicability of the proposed model, and the influence of different uncertainty adjustment parameters on capacity allocation was analyzed, demonstrating the unique advantages of the model in dealing with complex uncertainties.

2. Using upper and lower bounds to characterize the fluctuation range of wind power and load variables simplifies the representation of uncertainty excessively. This modeling approach often generalizes the variable fluctuation range, including events with extremely low probabilities that are unlikely to occur in actual operation. Since the goal of robust optimization is to ensure system stability under all possible scenarios, decision-making strategies become overly cautious in addressing such low-probability events. As a result, the system configuration becomes overly conservative.

3. The traditional box-type uncertainty set used in robust optimization describes uncertainty by defining the upper and lower bounds of fluctuation ranges, ensuring that optimization results are immune to the effects of variations in wind power and load parameters. However, the box-type uncertainty set proposed in this paper introduces an additional uncertainty adjustment parameter *Г* into the traditional model. This parameter plays a critical role in adjusting the extent to which uncertain variables deviate from their predicted values, thereby enabling a dynamic balance between economic performance and robustness. Specifically, by tuning the value of the uncertainty adjustment parameter *Г*, the number of time periods during which the system operates under the worst-case scenario can be controlled. The larger the value of the parameter *Г*, the more the total number of periods in the worst scenario is, the more conservative the system configuration scheme is, and the corresponding economy deteriorates.

4. Decision constraints have been given in Section 4.2 of this paper.

5. The data in Table 1 are derived from reference 14, the reference of hydrogen selling price is reference 15, the reference of penalty cost for lack of load is reference 16, and the reference of fluctuation deviation of electricity load and wind power output is reference 17.

6. **Revised in section 5.3 of the manuscript:**

The capacity of each device under different uncertainty adjustment parameters is shown in Fig 9 and Fig 10.

Fig 9.	The capacities of CHP units and wind power under different uncertainty adjustment parameters

Fig 10.	The capacities of ground source heat pumps, P2H equipment, and energy storage devices under different uncertainty adjustment parameters

From Fig 9 and Fig 10, it can be observed that the capacities of wind power and CHP units are relatively high, while the capacities of energy storage device, ground source heat pump, and P2H device are relatively low. Additionally, except for the P2H device, the capacities of other equipment increase with the rise of uncertainty parameters.

Fig 11.	Investment costs under different uncertainty adjustment parameters

The variation of investment costs with uncertainty parameters is shown in Fig 11. A comprehensive analysis with Figures 9 and 10 reveals the following: when the uncertainty parameter *Γ*=0, representing deterministic optimization, wind power and electric load remain stable and equal to the predicted values. In this case, the installed capacities of all system equipment, except for P2H devices, are minimal, resulting in the lowest investment cost. As the uncertainty adjustment parameter increases, the number of periods where wind power and electric load reach the interval boundaries rises, considering more uncertainties. Consequently, the storage capacity, ground source heat pump capacity, wind power capacity, and CHP unit capacity of the integrated electricity-heat-hydrogen energy system all increase, while the configured P2H device capacity decreases correspondingly, leading to a gradual rise in investment costs.

7. **Revised in the conclusion of the manuscript:**

To address the problems of wind power curtailment and operational reliability caused by the uncertainty of wind power and electricity demand and to improve the rationality of system capacity configuration, this paper conducts research on capacity optimization configuration of the electricity heat hydrogen regional integrated energy system in the context of dual-side uncertainties of supply and demand. The specific conclusions are as follows:

(1) Based on typical scenarios, a box-type uncertainty set independent of probability distribution is proposed to describe the uncertainty of wind power and load. By introducing uncertainty adjustment parameters, the reliability of the system is not only enhanced, but also flexible optimization space is provided for the economy of the system. With the adjustment of uncertainty adjustment parameters, the system can adaptively improve the reliability of power supply in different scenarios, especially in the case of large fluctuations in wind power and load, the system can ensure the stability of power supply and effectively reduce economic costs. The flexibility of this method allows it to adapt to a variety of uncertain situations and optimize decision outcomes.

(2) A two-stage robust optimization method considering the uncertainty of source load is proposed, and a min-max-min two-stage robust optimization multi-vector configuration model aiming at the minimum comprehensive system cost is established to optimize the unified configuration of wind power, CHP units, ground source heat pumps, P2H devices, energy storage and other equipment. Through the two-stage robust optimization method, the configuration of the system can not only adapt to the uncertainty of the source load, but also effectively reduce the impact of uncertainty on the operation of the integrated energy system. Compared with the configuration results of the deterministic optimal configuration model, the configuration results of the robust optimal configuration model have lower power purchase rate and LPSP in actual operation, demonstrating higher reliability.

(3) Models for CHP units, ground-source heat pumps, P2H units and energy storage devices are established. P2H units are jointly optimized with CHP units, wind power, ground-source heat pumps and energy storage devices in the integrated energy system to improve energy utilization efficiency, which can realize the complementarity and coordination of resources, reduce energy waste when the load of other equipment is excessive, improve the efficiency of equipment use, avoid equipment idling, and further reduce the overall operating cost. In the case of excess wind power, the P2H device can effectively convert wind curtailment into hydrogen energy, improve the efficiency of wind energy utilization and decrease wind curtailment rates.

**Response to Reviewer #2：**

1. In this paper, firstly, based on typical scenarios, a box-type uncertainty set independent of probability distribution is used to describe the uncertainty of wind power and load, forming a robust constraint. Then, with the goal of minimizing system investment and operating cost, the first stage is established as capacity configuration. The second stage is a two-stage min-max-min robust optimization configuration model running in the worst scenario. In this model, uncertain parameters are introduced to realize the trade-off between economy and robustness according to actual needs, and the conservative degree of configuration scheme is flexibly selected. Then, the model is derived and transformed according to the column and constraint generation algorithm and the strong duality theory, and the original problem is decomposed into linearized main problem and sub-problem for alternating solution. Finally, the reliability and applicability of the proposed model is verified by an integrated energy system in northern China, and the influence of different uncertainty adjustment parameters on capacity allocation is analyzed.

2. This figure is not blank, and there is a little purchased power at the 24th hour. The significance of the figure is to compare the two models under different sizes of prediction errors. By analyzing the data of power purchase, load power shortage, P2H device working state, etc., it is further explained that the robust optimal configuration model takes into account reliability and economy, and can achieve the best trade-off between economy and safety.

**Response to Reviewer #3：**

1. **Revised in the introduction of the manuscript:**

In summary, the following conclusions can be drawn:

The advantage of robust optimization lies in its ability to focus on the fluctuation boundaries of uncertain parameters without requiring a detailed understanding of their probabilistic distribution characteristics. It demonstrates strong resilience and is suitable for system environments where safety and reliability are the top priorities. However, to account for all possible disturbances, robust optimization may lead to relatively conservative decisions, potentially resulting in resource wastage and reduced benefits.

The advantage of interval optimization is that it only requires prior knowledge of the upper and lower bounds, or the midpoint and width, of the interval numbers. On one hand, obtaining these parameter values is typically easier. On the other hand, random variables can be converted into interval numbers through confidence levels or fuzzy numbers, thereby transforming stochastic optimization models directly into interval optimization models. This method can effectively handle uncertainty with known interval ranges and is applicable in cases where the uncertainty range of parameters in integrated energy systems is known but their specific probabilistic distributions are unclear. However, since interval optimization only considers uncertainties within the interval, the precision of its optimization results is often inferior to that of stochastic or robust optimization.

The advantage of stochastic optimization is its flexibility in handling highly random problems. It is well-suited for systems with uncertainties such as fluctuations in renewable energy generation and significant randomness in load forecasting. However, compared to other optimization methods, stochastic optimization requires substantial computational resources when addressing complex systems, leading to longer computation times and lower efficiency.

2. **Revised in the introduction of the manuscript:**

Therefore, based on the above research, this paper makes the following contributions:

(1) In order to solve the problem of optimal allocation of the electricity heat hydrogen regional integrated energy system, based on typical scenarios, a box-type uncertainty set independent of probability distribution is proposed to describe the uncertainty of wind power and demand, forming robust constraints.

(2) An innovative two-stage robust optimization model is constructed. The first stage of the model is capacity configuration, and the second stage considers the operation in the worst scenario, and adopts min-max-min structure to optimize the configuration. In order to flexibly adjust the degree of conservatism according to the actual demand, the uncertainty adjustment parameter *Г* is introduced into the model. By adjusting the parameter *Г*, the economy and feasibility can be more reasonably balanced, so as to provide higher adaptability for optimization in different scenarios.

(3) In order to further improve the solving efficiency of the model, the model is derived and transformed by combining the column and constraint generation algorithm and the strong duality theory, and the original problem is successfully decomposed into linearized main problem and subproblem, and efficient solution is achieved by alternate solving method.

(4) An integrated energy system in northern China was used to verify the reliability and applicability of the proposed model, and the influence of different uncertainty adjustment parameters on capacity allocation was analyzed, demonstrating the unique advantages of the model in dealing with complex uncertainties.

3. Temporal correlation in time series implies that variations in uncertainty may be sequential and influenced by previous moments. When adopting a box-type uncertainty set that considers temporal correlation, it is necessary to account for the regular patterns of wind power and load uncertainties over time. However, the box-type uncertainty set used in this study adjusts the total number of periods in which wind power and load uncertainties reach their worst-case scenarios within the uncertainty set U by introducing an uncertainty adjustment parameter. This enables a balance between reliability and economic efficiency based on system requirements, without considering the temporal variation patterns of wind power and load uncertainties. Therefore, the box-type uncertainty set established in this study does not incorporate temporal correlation.

4. The scenario considered in this paper is a regional integrated energy system comprising electricity, heat, and hydrogen. The system includes wind turbines, CHP units, ground-source heat pumps, P2H systems, and energy storage devices. The study focuses on the heating period of an integrated energy system in northern China, characterized by abundant wind and geothermal energy resources but relatively limited solar energy resources. Due to the system’s design being tailored for the dry and cold conditions of northern China, it is not suitable for humid and cold environments in southern regions. Consequently, no extensions to other environmental conditions are included in this paper.

5. **Revised in section 4.3 of the manuscript:**

Since the robust configuration model proposed in this paper is a non-convex optimization problem, the choice of solution algorithm must account for the ability to handle non-convex optimization. The column-and-constraint generation (C&CG) algorithm is well-suited for this purpose, as it decomposes the original non-convex optimization problem into a bilevel optimization problem consisting of a master problem and a subproblem, which are solved iteratively. This makes it an appropriate choice for addressing the proposed problem.

Additionally, this study compares the C&CG algorithm with the traditional Benders decomposition method. The results show that the C&CG algorithm converges within a shorter time frame, achieving faster and more efficient solutions. The specific reasons are as follows: (1) The C&CG algorithm narrows the solution space by rigorously filtering scenarios, thereby reducing the computational complexity. (2)When solving the master problem, the C&CG algorithm retains the original structural integrity of the model. In contrast, the Benders decomposition method primarily relies on dual cutting planes, which disrupt the original model structure and complicate the solution process. Therefore, this study employs the C&CG algorithm to solve the robust optimization configuration model for the electricity heat hydrogen regional integrated energy system.

6. **Revised in section 5.3 of the manuscript:**

The capacity of each device under different uncertainty adjustment parameters is shown in Fig 9 and Fig 10.

Fig 9.	The capacities of CHP units and wind power under different uncertainty adjustment parameters

Fig 10.	The capacities of ground source heat pumps, P2H equipment, and energy storage devices under different uncertainty adjustment parameters

From Fig 9 and Fig 10, it can be observed that the capacities of wind power and CHP units are relatively high, while the capacities of energy storage device, ground source heat pump, and P2H device are relatively low. Additionally, except for the P2H device, the capacities of other equipment increase with the rise of uncertainty parameters.

Fig 11.	Investment costs under different uncertainty adjustment parameters

The variation of investment costs with uncertainty parameters is shown in Fig 11. A comprehensive analysis with Fig 9 and Fig 10 reveals the following: when the uncertainty parameter *Γ*=0, representing deterministic optimization, wind power and electric load remain stable and equal to the predicted values. In this case, the installed capacities of all system equipment, except for P2H devices, are minimal, resulting in the lowest investment cost. As the uncertainty adjustment parameter increases, the number of periods where wind power and electric load reach the interval boundaries rises, considering more uncertainties. Consequently, the storage capacity, ground source heat pump capacity, wind power capacity, and CHP unit capacity of the integrated electricity-heat-hydrogen energy system all increase, while the configured P2H device capacity decreases correspondingly, leading to a gradual rise in investment costs.

7. **Revised in the conclusion of the manuscript:**

China's 14th Five-Year Plan emphasizes the green transition of the energy structure, promoting the development of low-carbon energy and encouraging the use of clean energy such as wind, solar, and hydrogen, while reducing reliance on traditional fossil fuels. The dual-carbon goals (carbon peaking and carbon neutrality) are particularly significant for northern China. This region has long depended on traditional energy sources like coal, making the transition to a greener energy structure a significant challenge.

In recent years, northern China's energy market has undergone substantial transformation in the development of renewable energy and a low-carbon economy. Air pollution and greenhouse gas emissions caused by coal combustion have become key drivers of energy structure reform in the region. During the winter heating season, the high demand for heating makes coal-fired heating the primary source of pollution and carbon emissions. Although the government has encouraged the development of green energy, coal still occupies a significant position in the supply and demand dynamics of the energy market.

The electricity heat hydrogen regional integrated energy system optimization method proposed in this study, takes northern China as a reference. It effectively enhances the integration and utilization of wind power, reduces wind curtailment, and minimizes the consumption of fossil fuels while enabling hydrogen production from curtailed wind power. By ensuring both economic viability and supply reliability, this method aligns well with the dual-carbon goals and provides a viable pathway for achieving them.

8. **Revised in the conclusion of the manuscript:**

On the basis of the existing research, this paper studies the capacity optimization of the integrated energy system in the electrothermal hydrogen region, but there are still some shortcomings. In the following aspects can also be further studied:

(1) The comprehensive energy system of electric hydrogen region has various forms of energy, and model establishment is the primary problem. The heat network model has the characteristics of time lag, so a more accurate mathematical model needs to be established, and the impact of time lag on heating can be further studied in the future.

(2) This paper only optimizes the equipment capacity of the integrated energy system in the electrothermal hydrogen region, without considering the flexibility of each equipment during the operation of the system. How to further combine the actual energy use scenario and add flexibility constraints into the model will be the next research focus.
